# Identification of major QTLs for soybean seed size and seed weight traits using a RIL population in different environments

**DOI:** 10.3389/fpls.2022.1094112

**Published:** 2023-01-11

**Authors:** Shilin Luo, Jia Jia, Riqian Liu, Ruqian Wei, Zhibin Guo, Zhandong Cai, Bo Chen, Fuwei Liang, Qiuju Xia, Hai Nian, Yanbo Cheng

**Affiliations:** ^1^ The State Key Laboratory for Conservation and Utilization of Subtropical Agro-bioresources, South China Agricultural University, Guangzhou, Guangdong, China; ^2^ The Key Laboratory of Plant Molecular Breeding of Guangdong Province, College of Agriculture, South China Agricultural University, Guangzhou, Guangdong, China; ^3^ Guangdong Laboratory for Lingnan Modern Agriculture, Guangzhou, Guangdong, China; ^4^ Rice Molecular Breeding Institute, Granlux Associated Grains, Shenzhen, Guangdong, China

**Keywords:** soybean, seed size, seed weight, stable QTLs, QTL clusters

## Abstract

**Introduction:**

The seed weight of soybean [*Glycine max* (L.) Merr.] is one of the major traits that determine soybean yield and is closely related to seed size. However, the genetic basis of the synergistic regulation of traits related to soybean yield is unclear.

**Methods:**

To understand the molecular genetic basis for the formation of soybean yield traits, the present study focused on QTLs mapping for seed size and weight traits in different environments and target genes mining.

**Results:**

A total of 85 QTLs associated with seed size and weight traits were identified using a recombinant inbred line (RIL) population developed from Guizao1×B13 (GB13). We also detected 18 environmentally stable QTLs. Of these, *qSL-3-1* was a novel QTL with a stable main effect associated with seed length. It was detected in all environments, three of which explained more than 10% of phenotypic variance (PV), with a maximum of 15.91%. In addition, *qSW-20-3* was a novel QTL with a stable main effect associated with seed width, which was identified in four environments. And the amount of phenotypic variance explained (PVE) varied from 9.22 to 21.93%. Five QTL clusters associated with both seed size and seed weight were summarized by QTL cluster identification. Fifteen candidate genes that may be involved in regulating soybean seed size and weight were also screened based on gene function annotation and GO enrichment analysis.

**Discussion:**

The results provide a biologically basic reference for understanding the formation of soybean seed size and weight traits.

## 1 Introduction

Soybean is one of the globally esteemed food crops with numerous nutritionalsubstances including protein, carbohydrates, lipids, minerals, vitamins, and bioactive substances such as isoflavones, saponins, sterols and phospholipids. With its vital values in agriculture and economy, soybean plays an important role in agricultural and industrial activities such as processing food and feed and producing textiles. While the demand for soybean has been increasing globally, soybean yield enhancement is now receiving significant attention for its potential for evolving productivity.

Seed size and seed weight are quantitative traits controlled by multiple genes and constrained by environmental factors. The forming of both traits is a complex biological process (Li et al., 2020). Seed size usually measure s seed length, seed width, and seed thickness (Salas et al., 2006). Many studies have been done worldwide on QTL localization of seed size and weight in soybean. In contrast, studies on QTL localization for seed size were relatively less than that for seed weight. For example, on the soybean data website SoyBase (http://www.soybase.org/) as of March 2022, the number of QTL publications are 18 on seed length, 16 on seed width, and 23 on seed thickness versus the number of QTL publications on seed weight are 300.

Salas et al. (2006) detected a total of 19 QTLs associated with seed shape using three RIL populations in two environments. Xu et al. (2011) used MAJ Multi-QTL Joint Analysis (MAJ) and composite interval mapping (CIM) for QTLs mapping. A total of 121 main QTLs were detected in the F_2:3_, F_2:4_ and F_2:5_ populations from the direct and reciprocal crosses of Lishuizhongzihuang with Nannong493-1. Sun et al. (2021) used a RIL population to detect QTLs for seed size traits in four environments. Ten QTLs controlling related traits were identified, of which five QTLs distributed on chromosomes 02, 04, 06, 13 and 16 were detected in at least two environments with PVE ranging from 3.6 to 9.4%. The results of similar studies (Salas et al., 2006; Xu et al., 2011; Hu et al., 2013; Niu et al., 2013; Yang et al., 2017; Teng et al., 2018; Gao et al., 2019; Cui et al., 2020; Sun et al., 2021; Kumawat and Xu, 2021) vary form different localization methods and different genetic backgrounds

The first study on QTL localization of seed weight in bean was reported by Sax (1923), in which he used seed colors as the markers. With the advancement of molecular marker technology, scholars had developed various molecular markers such as restriction fragment length polymorphism (RFLP), simple sequence repeat (SSR) and single nucleotide polymorphism (SNP) with different mapping populations to identify QTLs of soybean seed weight. In 1996, Maughan et al. (1996) detected 6 QTLs associated with seed weight in 150 F_2_ populations and their 150 F_2:3_ lines using 91 polymorphic genetic markers including RFP, RAPD and SSR. Zhang et al. (2016) performed a genome-wide association study (GWAS) using 309 soybean germplasm resources with 31045 SNP markers and associated QTLs related to seed weight to chromosome 04 and 19. More results from previous studies (Mian et al., 1996; Csanadi et al., 2001; Lee et al., 2001; Chapman et al., 2003; Fasoula et al., 2004; Hyten et al., 2004; D. Li et al., 2008; Han et al., 2012; Pathan et al., 2013; Kato et al., 2014; Yan et al., 2014; Kulkarni et al., 2017; Liu D. et al., 2018) are not identical, but these QTLs have laid a foundation for molecular marker-assisted breeding.

In summary, most researchers use different numbers of mapping populations to detect QTL in various environments. However, studies using more mapping populations had fewer experimental settings, in contrast to studies using fewer mapping populations tended to have more experimental environments. In this study, one mapping population is used to detect QTL in six environments suggesting a higher standard for the identification of stable QTL. In addition, the QTLs localization of soybean seed size and weight traits by RIL population in different environments and the mining of main effect genes can help us to understand the biological basis of soybean yield trait forming process, which has great theoretical and practical significance in guiding the molecular design breeding and in consistently enhancing soybean yield.

## 2 Materials and methods

### 2.1 Materials and planting

GB13 RIL population, comprising 248 lines, constructed with Guizao1 as the female parent and B13 as the male parent was planted on three replications in July 2018, July 2019 and July 2020 at the Teaching and Research Base of Zengcheng (23°14´N, 113°38´E), and in July 2019 and July 2020 at the Experimental Teaching Base of Guangzhou (23°10´N, 113°22´E) (designated as 18ZC, 19ZC, 20ZC, 19GZ and 20GZ, respectively). In addition, one F_1_ and 248 F_8:11_ RILs of the GB13 RIL population were developed using the single seed descent method (Jiang et al., 2019). The experimental materials were planted in a completely randomized zonal design with one row per strain, a row length of 2.0 m, a row spacing of 0.5 m, and a plant spacing of 0.1 m. Field management was carried out according to conventional standards, and no pest or disease outbreaks occurred during growth.

### 2.2 Phenotypic statistics and analysis

**Figure 1:**
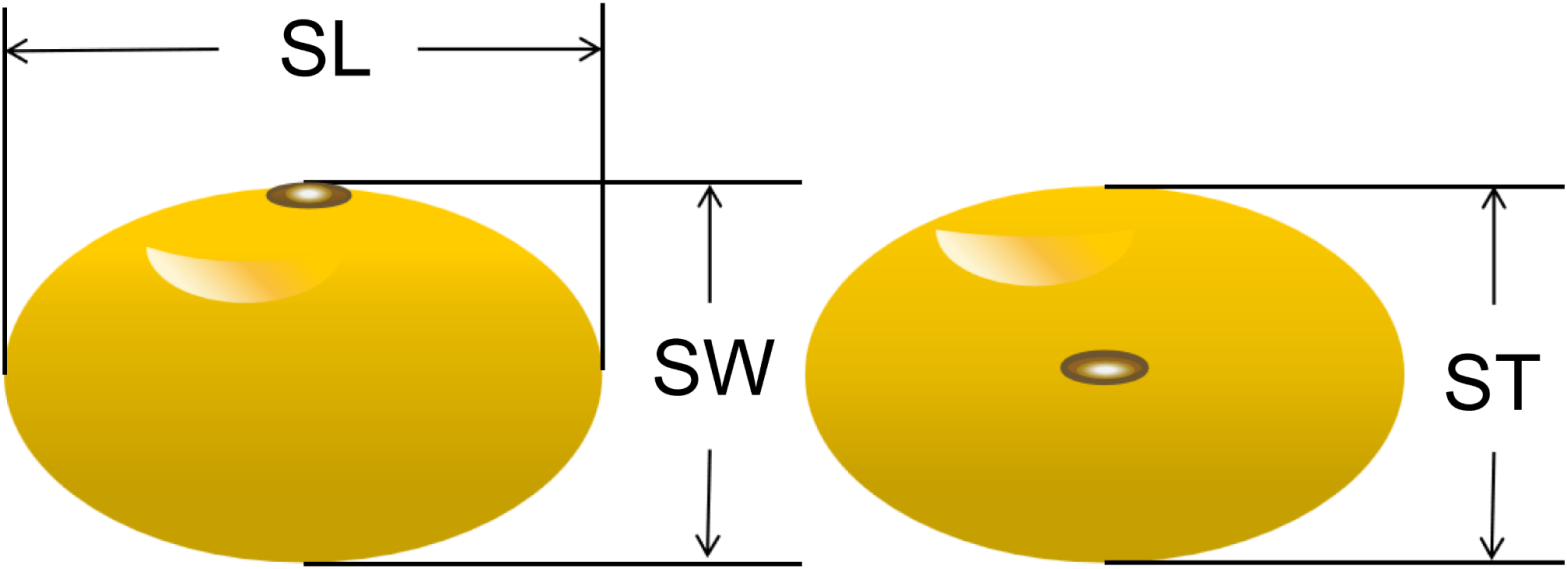
Measuring seed length (SL), seed width (SW) and seed thickness (ST).

Seed length (SL), seed width (SW) and seed thickness (ST) of soybean seeds were measured using an electronic vernier caliper (**Figure 1**). Twenty seeds from each strain were randomly selected for seed size measurement, after which they were weighed on an electronic balance. After three repetitions, five times the average of the weights were used as phenotypic data for the seed weight trait (100-seed weight, HSW).

The mean of the phenotypic data from the GB13 RIL population in five natural environments was calculated as the phenotypic data of the sixth environment (combined environment, designated as CE). Descriptive statistics, linear regression analysis and calculation of the skewness and the kurtosis were performed on phenotypic data from six environments using GraphPad Prism software (GraphPad Software Inc. v.7.0.4, United States), and the corresponding tables or figures were generated. Analysis of variance (ANOVA) and estimation of broad-sense heritability (*h* ^2^) were performed on phenotypic data from the five natural environments using Genstat 12_th_ Edition software. The formulae (Nyquist and Baker, 1991; Elattar et al., 2021) were as follows:


$$h^{2}=\sigma_{g}^{2}/(\sigma_{g}^{2}+\sigma_{ge}^{2}/n+\sigma_{e}^{2}/nr)$$

where σ^2^_g_ denotes genotype variance, σ^2^_ge_ denotes genotype × environment interaction variance, and σ^2^_e_ denotes variance error. “n” denotes the number of environments, and “r” denotes the number of replicates per environment.

### 2.3 QTL localization for seed size and weight traits and analysis

The composite interval mapping method (Zeng, 1994) was used to map QTLs with a high-density bin linkage map previously constructed in the laboratory, as described, the map contained 56,561 SNPs and 3715 bin markers covering a total genome length of 3049.2 cM, with the length of the linkage cluster ranging from 120.22-211.37 cM, The average distance between markers was 0.80 cM, and the number of bin markers on each cluster varied from 147 to 259. (Jiang et al., 2019). The additive effect (ADD) and PVE of each QTL were analyzed by the WinQTLCart 2.5 software. The threshold of LOD for different traits was set to 2.5 at the 5% significance level, and LOD thresholds greater than 2.5 were considered as the basis for determining the presence of QTLs. QTLs located on the same chromosome with adjacent marker intervals were combined into one QTL if their confidence intervals overlapped. QTLs were named according to “q + trait + chromosome + sequence number”, with the symbol “-” in between (Mccouch et al., 1997).

### 2.4 Stable QTLs and QTL clusters identification

Generally, QTLs detected in at least two environments were considered stable QTLs (Qi et al., 2017), but there were six experimental environments in this study, so QTLs detected in at least three environments were considered as stable QTLs in this study to identify the stable QTLs.

All QTLs were sorted by chromosome as the primary condition and physical position as the secondary condition. QTLs with identical chromosomes in overlapping or adjacent physical position (less than 5cM) were grouped into a cluster and identified as QTL cluster if it was associated with at least two traits (Liu et al., 2017; Wang et al., 2022). To mine the main effect and stable candidate genes, the identified QTL clusters contain at least one stable QTL in this study.

### 2.5 Candidate gene prediction

First, GO (Gene Ontology) term enrichment analysis was performed for the genes within the interval of the identified QTL clusters. This is useful for selecting candidate genes based on the sequence variation of genes between the parents. The GO annotation numbers were downloaded from the soybean data website SoyBase (https://www.soybase.org/), and then the WEGO2.0 online gene enrichment analysis mapping web page (http://wego.genomics.org.cn/) was used to create the gene enrichment cellular composition, molecular function and biological processes visualization diagrams. Next, genes that were highly or specifically expressed in soybean seeds were to be further screened. Gene expression data were obtained from the soybean data website SoyBase. Finally, the selected genes were functionally annotated by the Phytozome database (https://phytozome-next.jgi.doe.gov/), and then the candidate genes were identified based on the relevant literature and related gene functions. Meanwhile, the heat map of candidate genes expression was employed to analyze tissue-specific expression (Jia et al., 2022) by the online site (https://www.omicshare.com/tools/Home/Soft/heatmap), and the structures of candidate genes were mapped using the online site of the Gene Structure Display Server (http://gsds.gao-lab.org/index.php). The resequencing data of the parental lines were referred to compare the variation of candidate genes between parental lines. The whole genome of parental lines were sequenced using the Illumina HiSeq X Ten p, with an average sequencing depth of 8× (Xian et al., 2022). High-quality sequencing data were assessed to predict the gene structural variations.

## 3 Result

### 3.1 Correlation analysis between seed size and seed weight

**Figure 2:**
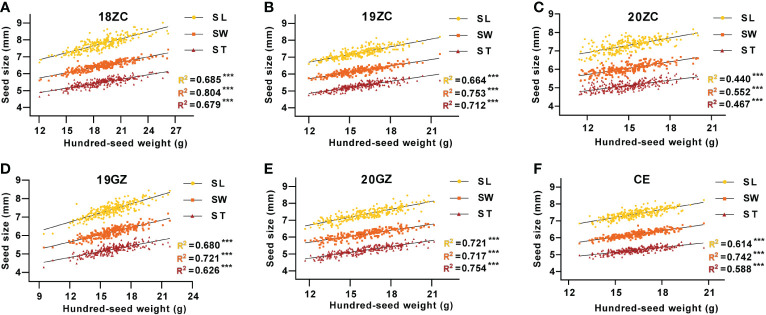
The correlation between seed size and seed weight of GB13 RIL population across the Teaching and Research Base of Zengcheng (ZC) in **(A)** 2018, **(B)** 2019 and **(C)** 2020, the Experimental Teaching Base of Guangzhou (GZ) in **(D)** 2019 and **(E)** 2020 and **(F)** their combined environment (CE). ***p < 0.001 (F-test). R^2^ indicates the correlation coefficient.

The regression linear analysis of seed size with seed weight shows that the SL, SW and ST were all highly significantly and positively correlated with HSW (*p*<0.01), with R-Squared (R^2^) of regression linear analysis ranging from 0.440 to 0.804 (**Figure 2**). In 18ZC, 19ZC, 19GZ, 20ZC and CE, the R^2^ between SW and HSW were the highest at 0.804, 0.721, 0.753, 0.552 and 0.742, respectively, while in 20GZ, the R^2^ between ST and HSW was the highest at 0.754. Thus, seed size is indeed closely related to seed weight. 18ZC, 19ZC and CE had the next largest R^2^ between SL and HSW at 0.685, 0.680 and 0.614, respectively and the smallest R^2^ between ST and HSW at 0.679, 0.626 and 0.588, respectively. 19GZ and 20ZC had the next largest R^2^ between ST and HSW at 0.713 and 0.467, and the smallest R^2^ between SL and HSW at 0.664 and 0.440. 20GZ had the next largest R^2^ between SL and HSW at 0.721, and the smallest R^2^ between SW and HSW at 0.717. Also, the above results suggest that the correlation between seed size and seed weight seems to be applied in field selection breeding as a reference for selecting high-yielding varieties.

### 3.2 Descriptive statistics analysis of seed size and weight traits

The phenotypic data were analyzed by descriptive statistics, and the results showed that the SL, SW, ST (**Supplementary Figure 1**) and HSW of the female parent Guizao1 were higher than those of the male parent B13 with significant differences (**Table 1**), which laid the foundation for QTL mapping. The GB13 RIL population differed remarkably insize and weight traits, both of which showed transgressive segregation. The coefficient of variation (CV) for SL ranged from 4.33 to 5.25%, for SW from 3.96 to 4.78%, for ST from 4.71 to 5.18%, and for HSW from 10.68 to 12.98%. The CV for HSW was greater than that for SL, SW and ST, but they were relatively stable, which indicated that both parents contained genes that were acting in the phenotypic variation.

**Table 1:** Statistical analysis ofseed size and weight traits for the parents and the lines at different environments.

Trait^#^	Env.^a^	Parents^b^		RILs^e^					
		Guizao1^c^	B13^d^	Range^f^	Mean^g^	SD^h^	CV (%)^i^	Skew.^j^	Kurt.^k^
SL	18ZC	8.34 ± 0.02	7.40 ± 0.01	6.66-9.01	7.77	0.41	5.25	0.12	-0.20
	19GZ	7.40 ± 0.02	6.90 ± 0.02	6.08-8.44	7.40	0.38	5.15	-0.19	0.66
	19ZC	7.40 ± 0.02	6.68 ± 0.02	6.52-8.21	7.27	0.32	4.33	0.32	-0.20
	20GZ	7.61 ± 0.01	6.86 ± 0.03	6.48-8.46	7.35	0.37	5.04	0.20	0.03
	20ZC	7.87 ± 0.03	6.85 ± 0.01	6.29-8.17	7.30	0.35	4.82	-0.35	-0.20
	CE	7.72 ± 0.07	6.94 ± 0.05	6.64-8.23	7.42	0.28	3.80	0.07	-0.03
SW	18ZC	6.65 ± 0.01	6.24 ± 0.01	5.59-7.43	6.46	0.29	4.44	-0.17	0.40
	19GZ	5.99 ± 0.02	5.84 ± 0.05	5.35-7.18	6.18	0.30	4.78	-0.01	0.22
	19ZC	6.02 ± 0.02	5.77 ± 0.02	5.65-7.18	6.20	0.25	3.96	0.31	0.46
	20GZ	5.97 ± 0.01	6.08 ± 0.02	5.37-6.95	6.18	0.28	4.55	-0.12	-0.23
	20ZC	6.10 ± 0.02	5.68 ± 0.03	5.37-6.75	6.05	0.27	4.50	-0.01	-0.27
	CE	6.34 ± 0.11	6.19 ± 0.08	5.66-6.85	6.21	0.21	3.39	0.14	-0.21
ST	18ZC	5.53 ± 0.01	5.22 ± 0.01	4.65-6.11	5.49	0.26	4.79	-0.22	0.07
	19GZ	5.17 ± 0.02	4.84 ± 0.04	4.28-5.90	5.24	0.25	4.83	-0.23	0.60
	19ZC	5.12 ± 0.01	4.90 ± 0.01	4.75-5.90	5.30	0.24	4.49	0.01	-0.47
	20GZ	5.11 ± 0.01	5.06 ± 0.01	4.52-5.88	5.23	0.27	5.18	-0.14	-0.39
	20ZC	5.22 ± 0.01	4.88 ± 0.02	4.61-5.81	5.12	0.24	4.71	0.25	-0.20
	CE	5.16 ± 0.01	5.05 ± 0.05	4.76-5.75	5.27	0.18	3.39	-0.10	-0.06
HSW	18ZC	18.85 ± 0.1	16.32 ± 0.06	12.05-26.22	18.88	2.45	12.98	0.14	0.28
	19GZ	15.35 ± 0.19	12.90 ± 0.24	9.45-21.80	16.09	1.91	11.89	0.02	0.61
	19ZC	14.97 ± 0.06	12.32 ± 0.05	12.00-21.63	15.77	1.68	10.68	0.29	0.02
	20GZ	15.40 ± 0.05	13.27 ± 0.13	11.70-21.17	16.08	2.01	12.52	0.14	-0.65
	20ZC	17.30 ± 0.12	13.02 ± 0.09	11.43-20.03	14.96	1.75	11.71	0.31	-0.17
	CE	16.37 ± 0.30	14.14 ± 0.51	12.70-20.70	16.35	1.41	8.62	0.15	-0.34

^#^Seed length (SL), seed width (SW), seed thickness (ST) and 100-seed weight (HSW).

a Environment.

b Parents of GB13 RIL population.

c Female parent of GB13 RIL population.

d Male parent of GB13 RIL population.

e Recombinant inbred lines.

f Range of seed size and 100-seed weight for GB13 RIL population.

g Mean of seed size and 100-seed weight for GB13 RIL population.

h Standard deviation.

i Coefficient of variation.

j Skewness.

k Kurtosis.

**Figure 3:**
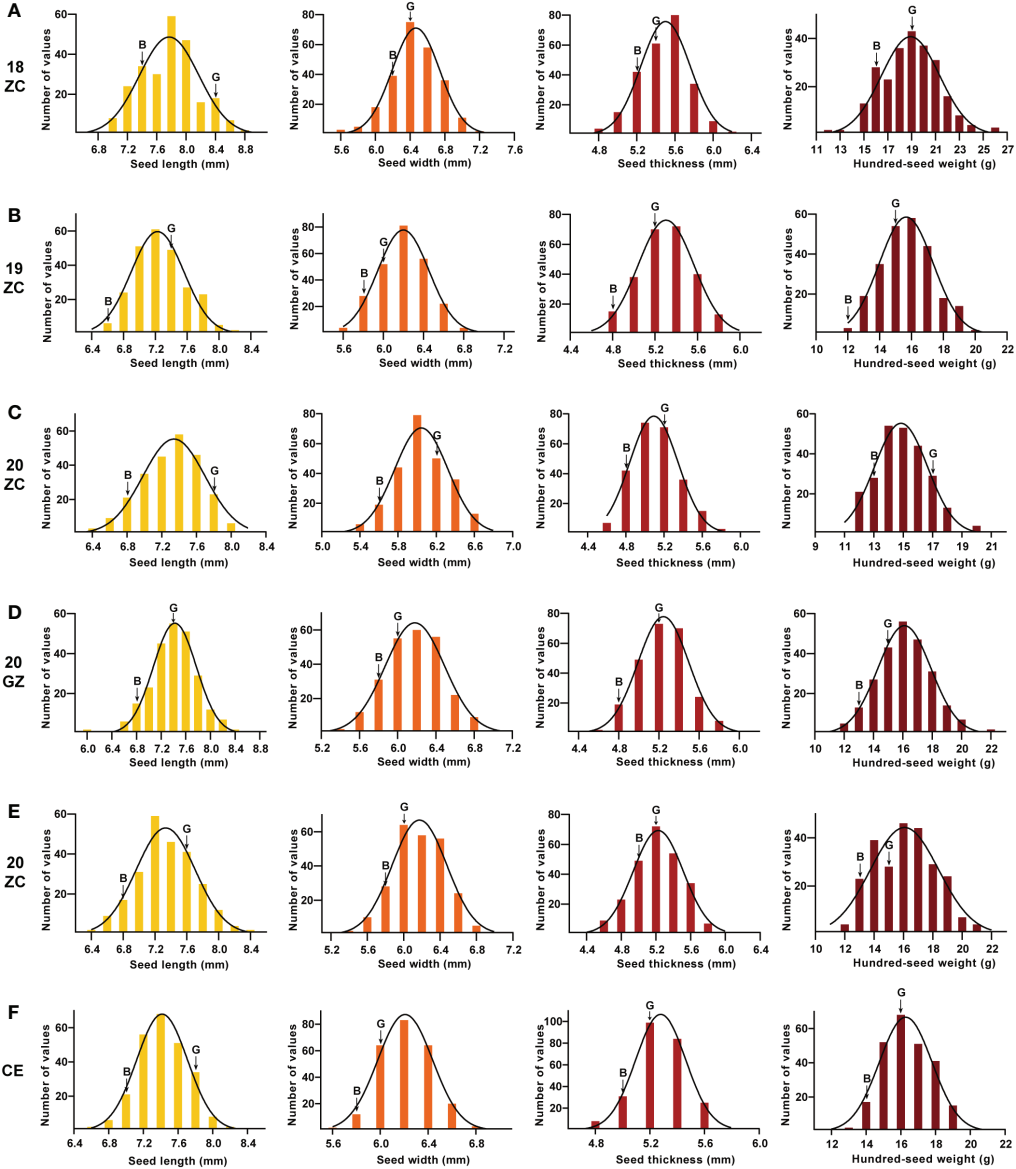
Frequency distribution and its fitted curve ofseed size and weight traits for GB13 RIL population across the Teaching and Research Base of Zengcheng (ZC) in **(A)** 2018, **(B)** 2019 and **(C)** 2020, the Experimental Teaching Base of Guangzhou (GZ) in **(D)** 2019 and **(E)** 2020 and **(F)** their combined environment (CE). The letter “G” indicates the female parent Guizao1, and the letter “B” indicates the male parent B13. The horizontal coordinates of the different colored figures from left to right were seed length, seed width, seed thickness and 100-seed weight.

The absolute values of the skewness and the kurtosis of SL, SW, ST and HSW of the GB13 RIL population were less than 1 (**Table 1**). In addition, the frequency distribution graph (**Figure 3**) showed that the phenotypic data of seed size and weight traits displayed continuous variation. The above results indicated that seed size and weight traits of the GB13 RIL population obeyed normal distribution, which was consistent with the characteristics of the RIL population and belonged to quantitative genetic traits.

### 3.3 Analysis of variance and estimates of broad-sense heritability

The results of ANOVA for SL, SW, ST and HSW of the GB13 RIL population in five natural environments (**Table 2**) show that the genotype, environment and interaction between genotype and environment had significant effects on seed size and weight traits of the GB13 RIL population (*p*<0.0001). The *h* ^2^ for seed size and weight traits of the GB13 RIL population were relatively high ranging from 0.74 to 0.83 (**Table 2**). The traits are suitable for further QTL localization analysis.

**Table 2:** Analysis of variance and broad-sense heritability for GB13 RIL population at five natural environments.

Trait^#^	Sources^a^	*Df* ^b^	*SS* ^c^	*MS* ^d^	*P* value^e^	VC^f^	PV (%)^g^	*h*² (%)^h^
SL	Repeat	2	0.74	0.37	<0.0001			0.83
	Genotype	247	294.11	1.19	<0.0001	0.07	46.49	
	Environment	4	123.42	30.86	<0.0001	0.04	19.51	
	Interaction	988	200.95	0.20	<0.0001	0.07	31.77	
	Error	2478	13.38	0.01				
	Total variation	3719	632.59					
SW	Repeat	2	1.09	0.54	<0.0001			0.82
	Genotype	247	164.87	0.67	<0.0001	0.04	45.42	
	Environment	4	66.05	16.51	<0.0001	0.02	18.19	
	Interaction	988	119.22	0.12	<0.0001	0.04	32.84	
	Error	2478	11.80	0.01				
	Total variation	3719	363.04					
ST	Repeat	2	1.09	0.54	<0.0001			0.74
	Genotype	247	118.51	0.48	<0.0001	0.02	38.23	
	Environment	4	54.34	13.58	<0.0001	0.02	17.53	
	Interaction	988	124.30	0.13	<0.0001	0.04	40.1	
	Error	2478	11.72	0.01				
	Total variation	3719	309.96					
HSW	Repeat	2	45.00	22.50	<0.0001			0.75
	Genotype	247	7210.17	29.19	<0.0001	1.46	33.32	
	Environment	4	6645.42	1661.36	<0.0001	2.22	30.71	
	Interaction	988	7256.28	7.34	<0.0001	2.38	33.54	
	Error	2478	479.53	0.19				
	Total variation	3719	21636.40					

^#^ Seed length (SL), seed width (SW), seed thickness (ST) and 100-seed weight (HSW).

a Sources of variation.

b Degree of freedom.

c Sum of deviation squares.

d mean square.

e The P value of the F-test (joint hypotheses test).

f Variance components for different sources of variation.

g Proportion of variation.

h Broad-sense heritability.

In terms of the proportion of total variation accounted for variation of different sources (**Table 2**), the largest source of variation in SL and SW was genotype, while the largest source of variation in ST and HSW was genotype × environment interactions. The most influenced by the environment was HSW, accounted for 30.71% of the total variation. It was followed by SL, SW and ST, which accounting for 19.51%, 18.19% and 17.53% of the total variation, respectively. The above results indicated that the seed weight trait was more influenced by the environment compared to the seed size trait.

### 3.4 Identification of stable QTL for seed size and weight traits

**Figure 4:**
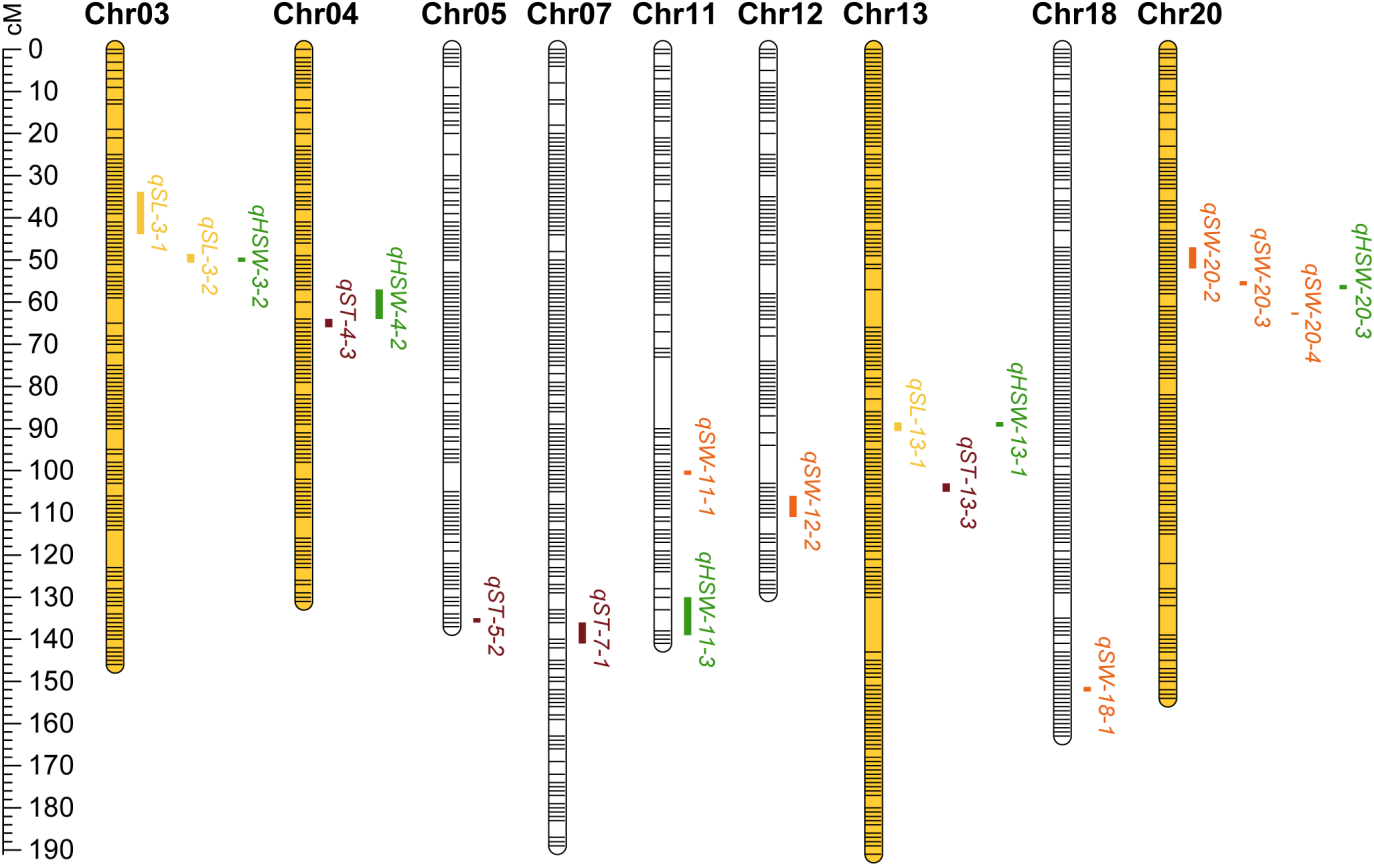
The linkage map of stable QTLs for soybean seed size and weight traits. The ruler on the left side indicates the interval distance between markers using cM (centimorgan) as the unit. Bars with yellow emphasis indicate the chromosome on which the QTL clusters are located. Between the bars in the graph, the yellow indicates QTL for SL; the orange indicates QTL for SW; the reddish brown indicates QTL for ST; the green indicates QTL for HSW.

A total of 85 QTL were detected for seed size and weight in six environments with PVE in the range of 3.14% - 21.93%, of which 19 QTLs were identified for SL, 23 for SW, 18 for ST, and 25 for HSW (**Supplementary Tables 1**–**4**). By collation and analysis, a total of 18 stable QTLs were identified (**Table 3**), including 3 for SL, 6 for SW, 4 for ST, and 5 for HSW (**Figure 4**). One QTL, *qSL-3-1*, associated with SL was detected in all environments, and it explained more than 10% of PV in three environments in the range of 11.93% - 15.91%. Three QTLs (*qHSW-11-3*, *qHSW-20-3* and *qSW-20-3*) were detected in four environments. Among them, *qSW-20-3* explained 9.22%, 10.79%, 20.89% and 21.93% of PV in four environments, respectively. Another 14 QTLs distributed on chromosomes 03, 04, 05, 07, 11, 12, 13, 18 and 20 were detected in three environments. In summary, integrating the PVE for QTL, the number of detections in various environments, and the relevant references, *qSL-3-1* and *qSW-20-3* can be considered as novel stable QTL with the main effects. Meanwhile, these stable QTLs above were important references for the excavation of genes controlling seed size and weight traits.

**Table 3:** Stable QTLs associated withseed size and weight traits for GB13 RIL population across different environments.

QTL^#^	Chr^a^	Interval	Position (cM)	PVE (%)^h^						LOD^i^						ADD^j^					
				E1^b^	E2^c^	E3^d^	E4^e^	E5^f^	E6^g^	E1	E2	E3	E4	E5	E6	E1	E2	E3	E4	E5	E6
*qSL-3-1*	*3*	bin32-bin43	43.1	13.57	6.03	11.93	4.38	6.61	15.91	9.37	4.03	7.49	3.15	4.61	11.45	0.15	0.09	0.11	0.08	0.09	0.11
*qSL-3-2*	*3*	bin51-bin55	49.4			12.15		9.07	14.56			7.63		6.42	10.38			0.11		0.11	0.11
*qHSW-3-2*	*3*	bin53-bin55	50.1			5.12		4.41	6.81			3.68		3.20	5.43			0.38		0.37	0.37
*qHSW-4-2*	*4*	bin75-bin78	57.5	5.34		4.68			5.28	3.75		3.25			4.18	0.59		0.37			0.33
*qST-4-3*	*4*	bin77-bin83	65.2	4.86	8.61				6.10	3.18	5.44				4.70	0.06	0.08				0.04
*qST-5-2*	*5*	bin155-bin158	135.1	4.67			4.52		3.69	3.06			3.13		2.89	-0.06			-0.06		-0.04
*qST-7-1*	*7*	bin159-bin161	139.9				3.85	5.75	6.99				2.69	4.06	5.37				0.05	0.06	0.05
*qSW-11-1*	*11*	bin95-bin100	101	3.89			7.48		6.34	2.90			5.23		5.14	-0.06			-0.08		-0.05
*qHSW-11-3*	*11*	bin131-bin134	133.1		4.90		8.26	3.33	4.41		2.99		5.76	2.56	3.41		0.42		0.59	0.32	0.31
*qSW-12-2*	*12*	bin103-bin109	111.1			5.79		3.69	5.39			4.37		2.83	4.36			0.06		0.05	0.05
*qHSW-13-1*	*13*	bin115-bin116	90.0		4.34			5.19	7.21		2.91			3.79	5.69		0.39			0.40	0.38
*qSL-13-1*	*13*	bin115-bin117	90	3.47				4.12	4.47	2.55				3.03	3.54	0.08				0.07	0.06
*qST-13-3*	*13*	bin143-bin149	104.6		5.09	5.52			4.56		3.27	3.74			3.50		0.06	0.06			0.04
*qSW-18-1*	*18*	bin237-bin239	152.4	5.62		4.91			3.91	4.03		3.72			3.24	0.07		0.05			0.04
*qSW-20-2*	*20*	bin46-bin49	49.1	7.46	11.80	8.64				5.30	8.29	6.24				-0.08	-0.10	-0.07			
*qSW-20-3*	*20*	bin64-bin68	55.3	9.22		10.79		21.93	20.89	6.63		7.89		15.04	15.24	-0.09	-0.10	-0.08		-0.14	-0.10
*qHSW-20-3*	*20*	bin68-bin73	55.9		6.12	3.31		4.68	7.21		4.06	2.52		3.42	5.70		-0.47	-0.31		-0.38	-0.38
*qSW-20-4*	*20*	bin84-bin86	64.2			3.80		7.30	5.81			2.65		4.56	3.86			-0.05		-0.09	-0.05

^#^The name of each QTL. ^a^Chromosome. ^b^at Zengcheng in 2018. ^c^at Guangzhou in 2019. ^d^at Zengcheng in 2019. ^e^at Guangzhou in 2020. ^f^at Zengcheng in 2020. ^g^Combined environment. ^h^Phenotypic variation explained. ^i^Log of odd value. ^j^Additive effect.

### 3.5 Identification of QTL clusters

According to the classification criteria of QTL clusters, we obtained five QTL clusters (**Table 4**) from the QTL mapping results (**Supplementary Tables 1**–**4**). These five QTL clusters were distributed on chromosomes 03, 04, 13 and 20. All of them were associated with HSW, which verified that the seed size was closely related to HSW from another aspect. The four QTL clusters related to SL were *qLH3-1*, *qLH3-2*, *qLWTH4* and *qLH13*; the two QTL clusters related to SW were *qLWTP4* and *qWTH20*; the two QTL clusters related to ST were *qLWTP4* and *qWTP20*. In terms of the number of traits controlled, one QTL cluster for four traits was *qLWTP4*, one QTL cluster for three traits was *qWTH20*, and three QTL clusters for two traits were *qLH3-1*, *qLH3-2*, and *qLH13*. Among them, *qLP3-1* and *qLP3-2* containing major and stable QTL(s) were located on the forearm of chromosome 03 at the physical positions between 4387438 and 6456915 bp and between 8784766 and 17199876 bp, respectively. Similarly, *qWTH20* also contained major and stable QTLs and was located at the physical position of chromosome 20 between 12056958 and 33222868 bp.

**Table 4:** QTL clusters associated with at least two traits.

Clusters	Chr^a^	Interval	Position(bp)	QTLs^b^	ADD^c^	PVE(%)^d^	Env.^e^
*qLH3-1*	3	bin32-bin43	4387438-6456915	*qSL-3-1*	0.15	13.57	18ZC
					0.09	6.03	19GZ
					0.11	11.93	19ZC
					0.08	4.38	20GZ
					0.09	6.61	20ZC
					0.11	15.91	CE
				*qHSW-3-1*	0.33	3.77	19ZC
					0.35	6.07	CE
*qLH3-2*	3	bin51-bin55	8784766-17199876	*qSL-3-2*	0.11	12.15	19ZC
					0.11	9.07	20ZC
					0.11	14.56	CE
				*qHSW-3-2*	0.38	5.12	19ZC
					0.37	4.41	20ZC
					0.37	6.81	CE
*qLWTH4*	4	bin74-bin83	9040807-12316836	*qSL-4-2*	0.06	4.36	CE
				*qSW-4-2*	0.06	3.78	18ZC
					0.04	3.70	CE
				*qST-4-3*	0.06	4.86	18ZC
					0.08	8.61	19GZ
					0.04	6.10	CE
				*qHSW-4-2*	0.59	5.34	18ZC
					0.37	4.68	19ZC
					0.33	5.28	CE
*qLH13*	13	bin115-bin117	27473391-27671175	*qSL-13-1*	0.08	3.47	18ZC
					0.07	4.12	20ZC
					0.06	4.47	CE
				*qHSW-13-1*	0.39	4.34	19GZ
					0.40	5.19	20ZC
					0.38	7.21	CE
*qWTH20*	20	bin64-bin73	27890104-33222868	*qSW-20-3*	-0.09	9.22	18ZC
					-0.08	10.79	19ZC
					-0.14	21.93	20ZC
					-0.10	20.89	CE
				*qST-20-2*	-0.05	7.55	CE
				*qHSW-20-3*	-0.47	6.12	19GZ
					-0.31	3.31	19ZC
					-0.38	4.68	20ZC
					-0.38	7.21	CE

^a^Chromosome. ^b^Indivadual QTLs of cluster. ^c^Additive effect. ^d^Phenotypic variation explained. ^e^Environment.

In summary, *qLP3-1*, *qLP3-2* and *qWTH20* may be QTL clusters containing major effect genes that regulate soybean seed size and weight traits; *qLWTP4* and *qLP13* may be QTL clusters containing micro-effector genes that harmonize the control of soybean seed size and weight traits. Therefore, these five QTL clusters can provide a reference for mining the target genes controlling seed size and weight traits, and were used as the target intervals for gene GO enrichment analysis.

### 3.6 Gene GO enrichment analysis

**Figure 5:**
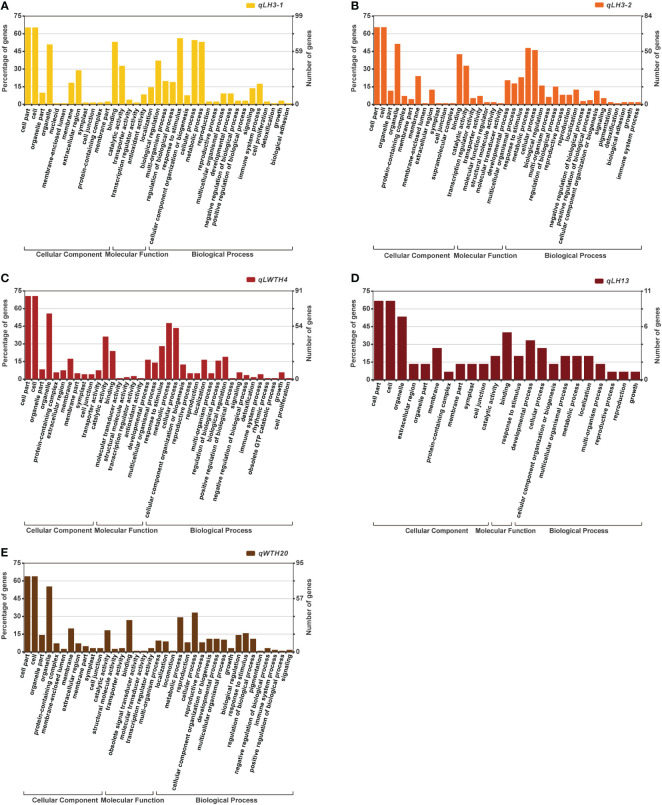
GO term enrichment analysis of the genes located within the five QTL clusters: **(A)** *qLH3-1*; **(B)** *qLH3-2*; **(C)** *qLWTH4*; **(D)** *qLH13*; **(E)** *qWTH20*.

By Gene GO enrichment analysis, we found that most of the genes within these five QTL cluster intervals were involved in cellular processes and metabolic processes (**Figure 5**). Most of the genes in the *qLP3-1* were involved in intracellular metabolic processes, bioregulation and reaction to stimuli, and were mostly involved in binding and catalytic activities in terms of molecular functions. While most of the genes in the *qLP3-2* were similar in function to those in the *qLP3-1*. In comparison, most of the genes in the *qLWTP4* were also involved in intracellular metabolic processes, but a small number of genes were involved in regulating growth and cell proliferation. The *qLP13* contained only 11 annotated genes, most of which were involved in growth and cellular processes. Similarly, most of the genes in the *qWTP20-2* were involved in intracellular metabolic processes.

### 3.7 Candidate genes in QTL clusters

Through the above gene GO enrichment analysis, followed by gene expression screening, gene function annotation, and sequence variation analysis, we obtained a total of 15 candidate genes (**Table 5**) that might be participating in the regulation of seed size and weight traits. Among them, six genes were located on chromosome 03, three on chromosome 04, two on chromosome 13, and four on chromosome 20. Among the 15 genes screened, *Glyma.03g040400* was the gene with the same function as the gene (*LOC_Os07g19000*) associated with seed size in rice; *Glyma.03g045400* and *Glyma.20g084000* were related to DNA methylation; *Glyma.04g100400*, *Glyma.20g081600* and *Glyma.20g087000* were hormone-related and might be involved in the regulation of seed size and weight traits by hormone signaling pathway; *Glyma.04g100100*, *Glyma.04g107100*, *Glyma.13g159500* and *Glyma.20g084500* might negatively regulate seed size and weight through the ubiquitin-proteasome pathway. *Glyma.03g036600* and *Glyma.03g065700* were associated with transcription factor regulation and might be involved in the regulation of seed size and weight traits through the transcription factor regulation pathway; *Glyma.03g044200*, *Glyma.03g065900* and *Glyma.13g160400* might be related to the metabolic synthesis of protein and oil, and indirectly regulated the seed size and weight traits.

**Table 5:** Candidate genes identified within the five QTL clusters that were highly expressed or specific expression in soybean seed.

Gene	Position (bp)	Gene functional annotation
*Glyma.03g036600*	4432910-4435002	Negative regulation of transcription; organ development; lipid transport calmodulin binding
*Glyma.03g040400*	5050463-5052307	Protease inhibitors (LTP family); Seed storage
*Glyma.03g044200*	5586338-5589780	Transporter protein activity; integral to membrane
*Glyma.03g045400*	5760642-5763461	Metabolic process; methyltransferase activity
*Glyma.03g065700*	11896538-11909845	Regulation of transcription; sequence-specific DNA binding transcription factor activity; DNA-dependent
*Glyma.03g065900*	12064850-12067709	Metabolic process; hydrolase activity; catalytic activity
*Glyma.04g100100*	9177207-9180239	Negative regulation of gene expression; response to abscisic acid stimulus; ubiquitin-protein ligase activity
*Glyma.04g100400*	9219564-9221412	Sterol biosynthetic process; response to oleuropein lactone stimulus
*Glyma.04g107100*	11239281-11250948	Regulation of meristem growth; Ubiquitin ligases are involved in synaptic protein degradation
*Glyma.13g159500*	27476780-27479031	Ubiquitin system components suggestive of protein; cell growth; cell morphogenesis
*Glyma.13g160400*	27606020-27608359	Lipid transport; lipid binding; Hydrophobic protein
*Glyma.20g081600*	30778586-30779164	Regulation of gene expression; response to auxin stimulus; protein binding
*Glyma.20g084000*	31536334-31538924	RNA methylation; RNA processing
*Glyma.20g084500*	31666190-31674959	Ubiquitin-protein ligase activity; protein ubiquitination; CUL4-RING ubiquitin ligase complex
*Glyma.20g087000*	32527435-32530216	Negative regulation of ethylene mediated signaling pathway; regulation of transcription; ethylene binding

**Figure 6:**
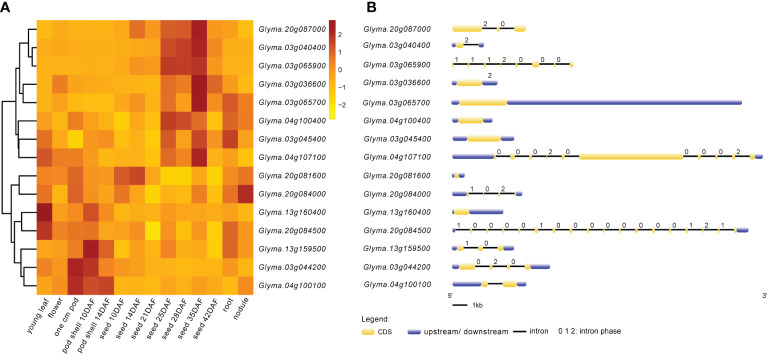
Expression patterns and structures of candidate genes. **(A)** Visual heat map of candidate genes expressed in different tissues and periods of soybean. The expression data of 15 candidate genes in this study were downloaded from SoyBase (https://www.soybase.org/soyseq/). Normalization of expression data by different genes. **(B)** Gene structure diagram of candidate genes. The yellow indicates CDS (coding DNA sequence); the blue indicates upstream or downstream of CDS containing 5’UTR and 3’UTR. Arabic numbers indicate the number of intron phases.

The expression heat map (**Figure 6A**) of these 15 candidate genes showed that two genes each were highly expressed in young leaf and nodule of soybean; three genes were specifically expressed at the pod shell development stage in soybean; eight genes including *Glyma.03g036600*, *Glyma.03g040400*, *Glyma.03g045400*, *Glyma.03g065700*, *Glyma.03g065900*, *Glyma.04g100400*, *Glyma.04g107100* and *Glyma.20g087000* were specifically and progressively expressed at the seed maturity stage in soybean. Meanwhile, the structural maps for most of the candidate genes (**Figure 6B**) were matched with their sequence in the parental lines, but ten candidate genes varied in the sequence of the parental lines, with the variant region on introns, 3’UTR (untranslated region) and 5’UTR (**Table 6**). They are *Glyma.03g036600*, *Glyma.03g040400*, *Glyma.03g044200*, *Glyma.03g045400*, *Glyma.03g065700*, *Glyma.04g100100*, *Glyma.04g107100*, *Glyma.13g160400*, *Glyma.20g084000* and *Glyma.20g084500*, and deserve to be investigated in depth.

**Table 6:** Sequence variations between the parental lines of candidate genes.

Gene	Chr^a^	Loci(bp)^b^	Guizao1^c^	B13^d^	Region^e^
*Glyma.03g036600*	3	4433324	T	TA	UTR3
*Glyma.03g040400*	3	5051148	A	ACG	intronic
	3	5051173	A	ATCACC	intronic
	3	5051386	T	TC	intronic
	3	5052301	G	GAT	UTR3
*Glyma.03g044200*	3	5588615	TCA	T	intronic
	3	5588681	AAAC	A	intronic
	3	5588699	AAC	A	intronic
*Glyma.03g045400*	3	5760781	T	TTC	UTR3
	3	5760989	TG	T	UTR3
	3	5761009	A	AAAAACAAGAG	UTR3
	3	5761114	AACTCTC	A	UTR3
	3	5763119	T	TATG	UTR5
*Glyma.03g065700*	3	11907032	G	GA	UTR3
*Glyma.03g065900*	3	NA	NA	NA	NA
*Glyma.04g100100*	4	9179539	G	GAAAACAAAAC	UTR5
*Glyma.04g100400*	4	NA	NA	NA	NA
*Glyma.04g107100*	4	11246102	GA	G	intronic
*Glyma.13g159500*	13	NA	NA	NA	NA
*Glyma.13g160400*	13	27607021	TA	T	UTR3
*Glyma.20g081600*	20	NA	NA	NA	NA
*Glyma.20g084000*	20	31536882	A	AAAAACCCTACATCTTCAT	intronic
	20	31537182	ATT	A	intronic
	20	31537333	TTA	T	intronic
	20	31537718	A	AGAATCAATGTCATT	intronic
	20	31538735	TTA	T	UTR3
*Glyma.20g084500*	20	31666328	GTCA	G	UTR3
	20	31669438	A	AAC	intronic
	20	31673766	TCG	T	intronic
	20	31674346	T	TAA	intronic
	20	31674567	T	TA	intronic
*Glyma.20g087000*	20	NA	NA	NA	NA

a Chromosome.

b Positions with variation in genes between parental lines.

c Female parent of GB13 RIL population.

d Male parent of GB13 RIL population.

e Region of variation in genes.

## 4 Discussion

Based on the results of the correlation analysis between seed size and seed weight in this study, the R2 between seed width and seed weight was the largest in most environments, so we suggest that varieties with wide seeds can be selected for varietal improvement for soybean seed weight trait. However, from the results of the QTLs localized for soybean seed size and weight traits, most of the QTLs for HSW overlapped or were close to the QTL interval for SL. It suggests that most of the genes with multi-effects may be genes that coordinate the control on both SL and HSW. Nevertheless, the specific regulatory mechanism is still unclear (He et al., 2021) and needs to be further investigated.

In this study, a total of 60 QTLs related to seed size trait and 25 QTLs related to seed weight trait were identified in the GB13 RIL population in five natural environments and their combined environment. Nineteen QTLs for SL included 4 stable QTLs and 15 sensitive QTLs (**Supplementary Table 1**); 23 QTLs for seed width included 6 stable QTLs and 17 sensitive QTLs (**Supplementary Table 2**); 18 QTLs for ST included 5 stable QTLs and 13 sensitive QTLs (**Supplementary Table 3**). The 25 QTLs associated with HSW included 5 stable QTLs and 20 sensitive QTLs (**Supplementary Table 4**). By comparing the results with those of previous studies, ten sensitive QTLs for SL (*qSL-2-1*, *qSL-3-3*, *qSL-4-1*, *qSL-4-2*, *qSL-4-3*, *qSL-6-2*, *qSL-11-1*, *qSL-11-2*, *qSL-18-2* and *qSL-20-1*) overlapped with the intervals of previous localization results or were within their subsets (Salas et al., 2006; Xu et al., 2011; Niu et al., 2013; Yang et al., 2017; Cui et al., 2020; Hina et al., 2020; Kumawat and Xu, 2021). Eight sensitive QTLs for SW (*qSW-2-1*, *qSW-2-2*, *qSW-4-1*, *qSW-9-1*, *qSW-10-1*, *qSW-15-1*, *qSW-20-1*, and *qSW-20-5*) overlap with or were within a subset of the previous localization result intervals (Xu et al., 2011; Niu et al., 2013; Yang et al., 2017; Hina et al., 2020). Five sensitive QTLs for ST (*qST-4-4*, *qST-5-2*, *qST-6-1*, *qST-13-1* and *qST-20-3*) overlapped with or included the intervals of previous localization results (Xu et al., 2011; Niu et al., 2013; Hina et al., 2020). Five sensitive QTLs for HSW (*qHSW-4-3*, *qHSW-13-2*, *qHSW-17-2*, *qHSW-20-1*, and *qHSW-20-5*) were found to have regions of overlap with the localization results of previous studies (Mansur et al., 1993; Teng et al., 2009; Han et al., 2012; Kato et al., 2014). Overall, the results of the present study correlated well with those of previous studies.

Besides, comparing the localization results of seed size traits in this study with the results of other related trait localization studies, eight QTLs (*qSL-4-1*, *qSL-17-1*, *qSL-20-1*, *qSW-12-2*, *qSW-15-2*, *qSW-17-1*, *qST-13-3*, *qST-20-1*) were found to overlap or interval overlap with QTLs related to yield traits from previous studies (Mansur et al., 1993; Kabelka et al., 2004; Du et al., 2009; Palomeque et al., 2009; Liu et al., 2011; Wang X. et al., 2014). Fifteen QTLs (*qSL-6-2*, *qSL-13-1*, *qSL-17-1*, *qSL-18-1*, *qSL-20-1*, *qSW-11-1*, *qSW-12-1*, *qSW-12-2*, *qSW-20-2*, *qSW-20-3*, *qST-5-2*, *qST-13-1*, *qST-13-3*, *qST20-1*, *qST-20-2*) overlapped QTLs associated with traits related to protein, oil and fatty acids (Sebolt et al., 2000; Tajuddin et al., 2003; Bachlava et al., 2009; Qi et al., 2011; Eskandari et al., 2013; Mao et al., 2013; Wang J. et al., 2014; Fan et al., 2015; Warrington et al., 2015; Han et al., 2015; Leite et al., 2016; Fliege et al., 2022). Five QTLs (*qSW-15-1*, *qSW-15-3*, *qSW-18-2*, *qST-4-1* and *qSW-5-2*) had overlapping intervals with QTLs related to seed-set of pod number and soybean maturity (Yao et al., 2015; Ning et al., 2018). Two QTLs (*qSL-5-1* and *qST-5-2*) had interval overlapping with isoflavonoid-related QTLs (Yang et al., 2011; Cai et al., 2018). The *qSW-4-1* overlapped with the QTLs associated with the long juvenility (Yue et al., 2017). Researchers sometimes studied the seed weight of soybean for QTLs mapping in collaboration with protein and oil content (Fasoula et al., 2004; Pathan et al., 2013; Yesudas et al., 2013; Wang X. et al., 2014; Whiting et al., 2020; He et al., 2021). In this study, *qHSW-13-1*, *qHSW-15-1*, *qHSW-17-2*, *qHSW-20-2* and the stable QTL *qHSW-20-3* had overlap intervals with QTLs related to protein or oil content studied previously (Panthee et al., 2005; Mao et al., 2013; Wang X. et al., 2014; Han et al., 2015; Fliege et al., 2022). Besides, *qHSW-15-1* and *qHSW-17-1* also had overlap intervals with yield-related QTL studied previously (Palomeque et al., 2009; Liu et al., 2011). In summary, many QTLs with stable main effects related to seed size and weight traits in this study were consistent with previous studies results and correlated with QTLs related to other traits including protein, oil content, pod number, yield, maturity, and long juvenile stage. Thus, we suggest that genes regulating soybean seed size and weight traits may be correlated with genes regulating other traits, especially protein and oil content, so genes regulating the synthesis or metabolism of protein and oil content were also used as the basis for candidate gene speculation in this study.

In addition, compared with soybean, rice (*Oryza sativa L.*) has been studied much more intensively than soybean in terms of its seed size and weight. Currently, genes associated with seed size and weight, such as *GW2* (Song et al., 2007), *OsGRF4* (Li et al., 2016), *GS3* (Fan et al., 2006), *qSW5* (Shomura et al., 2008), *GW5* (Weng et al., 2008), *qGL3* (Zhang et al., 2012), *LGY3* (Liu Q. et al., 2018), *GS5* (Li and Li, 2016), *GS6* (Sun et al., 2013), *GLW7* (Wang et al., 2015), etc., have been cloned. Furthermore, the regulatory pathways of the genes mainly include the ubiquitin-proteasome pathway, G protein signaling pathway, Mitogen-activated protein kinases (MAPK) signaling pathway, hormone signaling pathway and transcription factor regulatory pathway, etc. (Li and Li, 2016). Among them, in the ubiquitin-proteasome pathway, E3 ubiquitin ligase plays a major role and negatively regulates seed width and seed weight (Song et al., 2007). In the hormone signaling pathway, Brassinolide (BR) and Auxin (IAA) can promote cell growth and expansion (Li et al., 2019), it mainly regulates glume development to positively regulate seed size in rice (Li et al., 2011). Therefore, in this study, candidate genes were selected and predicted by screening genes within five clusters based on their specific expression in soybean, combined with homologous or identical functions of genes related to grain size and weight traits in rice.

The major and stable QTL, *qSW-20-3*, which was detected in four environments with PVE varied from 9.22 to 21.93%, was contained in the *qWTP20-2* cluster. By the analysis of sequence variation, we found that among the candidate genes within this QTL interval, only *Glyma.20g084000* and *Glyma.20g084500* were different in the sequence of the parental lines. They were not specifically expressed at the seed maturation stage in soybean, moreover, they have a negative additive effect, but they were associated with RNA methylation and protein ubiquitination, respectively. Therefore, we speculate that the genes within this cluster may indirectly regulate seed size and weight by regulating other traits. While another major and stable QTL, *qSL-3-1*, which was detected in six environments with PVE varied from 4.38 to 15.91%, was contained in the *qLP3-1* cluster. Comparing the sequence variation between parental lines for candidate genes within this cluster, we found that five genes underwent natural variation between parental lines, and all of them were specifically and highly expressed at the seed maturation stage in soybean. Moreover, they have a positive additive effect. Therefore, we speculate that the five candidate genes within the *qLP3-1* cluster regulate seed size and weight positively through certain pathways based on their gene annotation. However, the exact signaling pathway remains to be further investigated.

It is important to study the molecular regulatory network of yield-related traits in soybean. And the loci and candidate genes in this study provide an important theoretical basis and genetic resources for solving the bottleneck problem of soybean yield, which deserves to be further investigated at the molecular level.

## Data availability statement

The original contributions presented in the study are included in the article/**Supplementary Material**. Further inquiries can be directed to the corresponding authors.

## Author contributions

YC and SL designed the project. SL performed the experiments and drafted the manuscript. RL, ZG, BC, and FL helped the experiment to obtain part of the data. JJ and RW revised the manuscript. SL and QX analyzed the data. HN and ZC validated the manuscripts.
