# Supplementary figures and images for: Identification of major QTLs for soybean seed size and seed weight traits using a RIL population in different environments
Source: Front Plant Sci. 2023 Jan 11;13:1094112. doi: 10.3389/fpls.2022.1094112 (PMC9874164; doi:10.3389/fpls.2022.1094112)

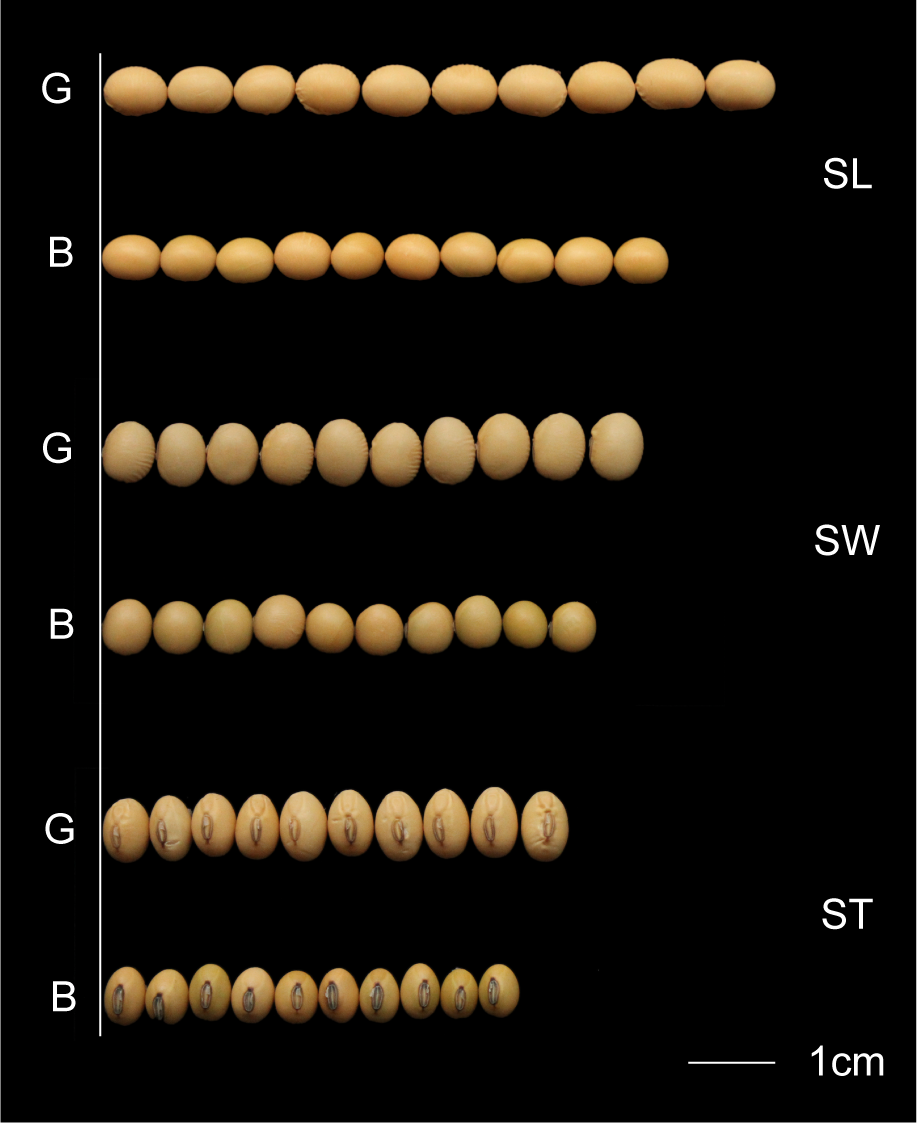

Supplement: Supplementary file 1 [file Image_1.tif]
