# Supplementary material for: Identification of major QTLs for soybean seed size and seed weight traits using a RIL population in different environments
Source: Front Plant Sci. 2023 Jan 11;13:1094112. doi: 10.3389/fpls.2022.1094112 (PMC9874164; doi:10.3389/fpls.2022.1094112)
Supplement: Supplementary file 2 [file Table_1.docx]

Supplementary Figure 1 The phenotype diagram for seed length (SL), seed width (SW) and seed thickness (ST) of parent lines. The letter "G" indicates the female parent Guizao1, and the letter "B" indicates the male parent B13.

Supplementary Table 1 QTLs identified for seed length trait in GB13 RIL across different environments

| QTL | Chr^a^ | Interval | Position (bp) | Position (cM) | LOD^b^ | ADD^c^ | PVE (%)^d^ | Env.^e^ | References |
| --- | --- | --- | --- | --- | --- | --- | --- | --- | --- |
| *qSL-2-1* | 2 | bin143-bin145 | 39848223-39987461 | 130.2 | 2.95 | -0.08 | 4.34 | 19GZ | （Kumawat et al. 2021） |
| *qSL-3-1* | 3 | bin32-bin43 | 4387438-6456915 | 35.3 | 9.37 | 0.15 | 13.57 | 18ZC |  |
|  |  |  |  | 38.4 | 4.61 | 0.09 | 6.61 | 20ZC |  |
|  |  |  |  | 43.1 | 7.49 | 0.11 | 11.93 | 19ZC |  |
|  |  |  |  | 43.1 | 4.03 | 0.09 | 6.03 | 19GZ |  |
|  |  |  |  | 43.1 | 11.45 | 0.11 | 15.91 | CE |  |
|  |  |  |  | 45 | 3.15 | 0.08 | 4.38 | 20GZ |  |
| *qSL-3-2* | 3 | bin51-bin55 | 8784766-17199876 | 49.4 | 6.42 | 0.11 | 9.07 | 20ZC |  |
|  |  |  |  | 49.4 | 10.38 | 0.11 | 14.56 | CE |  |
|  |  |  |  | 50.6 | 7.63 | 0.11 | 12.15 | 19ZC |  |
| *qSL-3-3* | 3 | bin75-bin77 | 31674416-33137748 | 59.2 | 3.51 | 0.08 | 4.86 | 20GZ | （Cui et al. 2020） |
| *qSL-4-1* | 4 | bin39-bin41 | 5744948-5838921 | 34.7 | 3.65 | 0.08 | 4.97 | 20ZC | （Yang et al. 2017） |
| *qSL-4-2* | 4 | bin74-bin76 | 9040807-9546369 | 57.5 | 3.44 | 0.06 | 4.36 | CE | （Salas et al. 2006；Hina et al. 2020） |
| *qSL-4-3* | 4 | bin162-bin164 | 49786918-49996727 | 116.4 | 3.27 | 0.09 | 4.46 | 18ZC | （Cui et al. 2020） |
| *qSL-5-1* | 5 | bin100-bin102 | 34370060-34917661 | 90.3 | 2.94 | 0.06 | 3.9 | 19ZC |  |

Supplementary Table 1 continued

| QTL | Chr^a^ | Interval | Position (bp) | Position (cM) | LOD^b^ | ADD^c^ | PVE (%)^d^ | Env.^e^ | References |
| --- | --- | --- | --- | --- | --- | --- | --- | --- | --- |
| *qSL-6-1* | 6 | bin1-bin2 | 1-989938 | 0 | 4.12 | 0.09 | 5.79 | 20GZ |  |
| *qSL-6-2* | 6 | bin31-bin33 | 4933392-5729762 | 38.1 | 2.72 | 0.05 | 3.51 | CE | （Xu et al. 2011；Hina et al. 2020） |
| *qSL-8-1* | 8 | bin147-bin149 | 39509026-42016655 | 128.1 | 2.81 | 0.08 | 3.84 | 18ZC |  |
| *qSL-11-1* | 11 | bin106-bin108 | 27762029-28460697 | 104.7 | 4.08 | -0.09 | 5.66 | 20GZ | （Cui et al. 2020） |
| *qSL-11-2* | 11 | bin134-bin135 | 33570965-33658063 | 139.7 | 3.58 | 0.08 | 5.04 | 20GZ | （Niu et al. 2013） |
| *qSL-13-1* | 13 | bin115-bin117 | 27473391-27671175 | 90 | 2.55 | 0.08 | 3.47 | 18ZC |  |
|  |  |  |  | 90 | 3.03 | 0.07 | 4.12 | 20ZC |  |
|  |  |  |  | 90 | 3.54 | 0.06 | 4.47 | CE |  |
| *qSL-17-1* | 17 | bin8-bin10 | 1909481-2137276 | 16.2 | 3.03 | 0.07 | 4.09 | 20ZC |  |
| *qSL-17-2* | 17 | bin49-bin51 | 7320307-8459319 | 54.2 | 3.44 | 0.06 | 4.36 | CE |  |
| *qSL-18-1* | 18 | bin19-bin21 | 2460215-2936665 | 13 | 4.58 | 0.09 | 6.45 | 20GZ |  |
| *qSL-18-2* | 18 | bin29-bin30 | 3254606-3692649 | 18.5 | 4.77 | 0.1 | 6.69 | 20GZ | （Xu et al. 2011） |
| *qSL-20-1* | 20 | bin153-bin162 | 41990737-44864841 | 129.5 | 2.71 | 0.05 | 3.22 | CE | （Kumawat et al. 2021） |
|  |  |  |  | 139.7 | 2.68 | 0.06 | 3.56 | 19ZC |  |

^a^Chromosome. ^b^The log of odds (LOD) value at the peak likelihood of QTL. ^d^Phenotypic variation explained. ^e^Environment.

Supplementary Table 2 QTLs identified for seed width trait in GB13 RIL across different environments

| QTL | Chr^a^ | Interval | Position (bp) | Position (cM) | LOD^b^ | ADD^c^ | PVE (%)^d^ | Env.^e^ | References |
| --- | --- | --- | --- | --- | --- | --- | --- | --- | --- |
| *qSW-2-1* | 2 | bin97-bin101 | 14922813-15587115 | 93.9 | 2.74 | -0.04 | 3.14 | CE | （Niu et al. 2013；Yang et al. 2017） |
|  |  |  |  | 100.8 | 3.4 | -0.07 | 4.93 | 19GZ |  |
| *qSW-2-2* | 2 | bin157-bin159 | 41365389-41705254 | 141.4 | 3.34 | -0.06 | 4.53 | 18ZC | （Hina et al. 2020） |
| *qSW-4-1* | 4 | bin19-bin19 | 2707907-4097410 | 24.2 | 3.75 | -0.07 | 5.19 | 19GZ | （Yang et al. 2017） |
| *qSW-4-2* | 4 | bin77-bin79 | 9159728-10115048 | 64.4 | 2.97 | 0.06 | 3.78 | 18ZC |  |
|  |  |  |  | 64.4 | 3.24 | 0.04 | 3.7 | CE |  |
| *qSW-9-1* | 9 | bin14-bin16 | 3371562-3979304 | 24.9 | 2.76 | 0.04 | 3.43 | CE | （Hina et al. 2020） |
| *qSW-10-1* | 10 | bin29-bin31 | 3306301-3873671 | 20.5 | 2.7 | -0.05 | 3.34 | 19ZC | （Xu et al. 2011） |
| *qSW-11-1* | 11 | bin95-bin100 | 15151676-18213414 | 100.1 | 5.14 | -0.05 | 6.34 | CE |  |
|  |  |  |  | 101 | 5.23 | -0.08 | 7.48 | 20GZ |  |
|  |  |  |  | 101.7 | 2.9 | -0.06 | 3.89 | 18ZC |  |
| *qSW-11-2* | 11 | bin108-bin110 | 28193727-29598942 | 106.1 | 4.73 | -0.07 | 6.8 | 20GZ |  |
| *qSW-12-1* | 12 | bin95-bin97 | 33432791-35013253 | 93.7 | 4.35 | 0.05 | 6.4 | CE |  |
| *qSW-12-2* | 12 | bin103-bin109 | 35464404-36328576 | 106 | 4.37 | 0.06 | 5.79 | 19ZC |  |
|  |  |  |  | 111.1 | 2.83 | 0.05 | 3.69 | 20ZC |  |
|  |  |  |  |  | 4.36 | 0.05 | 5.39 | CE |  |
| *qSW-14-1* | 14 | bin118-bin120 | 32052002-32660037 | 116.2 | 3.12 | -0.04 | 3.86 | CE |  |
| *qSW-14-2* | 14 | bin148-bin150 | 42605675-42819236 | 129.3 | 3.97 | -0.05 | 4.88 | CE |  |
| *qSW-15-1* | 15 | bin16-bin18 | 3955219-4264223 | 22.8 | 2.87 | 0.05 | 3.81 | 19ZC | （Yang et al. 2017） |
| *qSW-15-2* | 15 | bin24-bin26 | 4706262-5531628 | 28 | 2.9 | 0.06 | 3.97 | 19GZ |  |

Supplementary Table 2 continued

| QTL | Chr^a^ | Interval | Position (bp) | Position (cM) | LOD^b^ | ADD^c^ | PVE (%)^d^ | Env.^e^ | References |
| --- | --- | --- | --- | --- | --- | --- | --- | --- | --- |
| *qSW-15-3* | 15 | bin29-bin31 | 5944971-7199234 | 34.2 | 3.59 | 0.06 | 4.74 | 19ZC |  |
| *qSW-17-1* | 17 | bin40-bin42 | 4938708-5526049 | 44.2 | 2.79 | 0.04 | 3.52 | CE |  |
| *qSW-18-1* | 18 | bin237-bin239 | 55133917-55281850 | 152.4 | 3.72 | 0.05 | 4.91 | 19ZC |  |
|  |  |  |  |  | 3.24 | 0.04 | 3.91 | CE |  |
|  |  |  |  | 153 | 4.03 | 0.07 | 5.62 | 18ZC |  |
| *qSW-18-2* | 18 | bin250-bin252 | 56079924-56416403 | 158 | 5.28 | 0.08 | 7.3 | 18ZC |  |
|  |  |  |  |  | 3.68 | 0.05 | 4.86 | 19ZC |  |
| *qSW-20-1* | 20 | bin37-bin39 | 3056426-3144885 | 44.8 | 4.78 | -0.07 | 6.77 | 20GZ | （Yang et al. 2017） |
| *qSW-20-2* | 20 | bin46-bin53 | 5369281-12095409 | 47.5 | 6.24 | -0.07 | 8.64 | 19ZC |  |
|  |  |  |  | 49.1 | 5.3 | -0.08 | 7.46 | 18ZC |  |
|  |  |  |  | 51.5 | 8.29 | -0.1 | 11.8 | 19GZ |  |
| *qSW-20-3* | 20 | Bin64-bin68 | 27890104-31573673 | 55.3 | 15.04 | -0.14 | 21.93 | 20ZC |  |
|  |  |  |  | 55.3 | 15.24 | -0.1 | 20.89 | CE |  |
|  |  |  |  | 55.9 | 6.63 | -0.09 | 9.22 | 18ZC |  |
|  |  |  |  | 55.9 | 7.89 | -0.08 | 10.79 | 19ZC |  |
| *qSW-20-4* | 20 | bin84-bin86 | 34383022-34599659 | 64.2 | 2.65 | -0.05 | 3.8 | 19ZC |  |
|  |  |  |  | 64.2 | 4.56 | -0.09 | 7.3 | 20ZC |  |
|  |  |  |  | 64.2 | 3.86 | -0.05 | 5.81 | CE |  |
| *qSW-20-5* | 20 | bin142-bin146 | 38972351-41472016 | 114.1 | 5.15 | 0.08 | 7.04 | 18ZC | （Xu et al. 2011） |
|  |  |  |  | 121.6 | 2.67 | 0.04 | 3.42 | CE |  |

^a^Chromosome. ^b^The log of odds (LOD) value at the peak likelihood of QTL. ^d^Phenotypic variation explained. ^e^Environment.

Supplementary Table 3 QTLs identified for seed thickness trait in GB13 RIL across different environments

| QTL | Chr^a^ | Interval | Position (bp) | Position (cM) | LOD^b^ | ADD^c^ | PVE (%)^d^ | Env.^e^ | References |
| --- | --- | --- | --- | --- | --- | --- | --- | --- | --- |
| *qST-4-1* | 4 | bin14-bin16 | 3371562-3979304 | 20.3 | 6.71 | 0.08 | 9.6 | 20ZC |  |
| *qST-4-2* | 4 | bin31-bin33 | 4820821-5222780 | 28.5 | 5.39 | 0.07 | 7.4 | 20ZC |  |
| *qST-4-3* | 4 | bin77-bin83 | 9546370-12316836 | 63.6 | 5.44 | 0.08 | 8.61 | 19GZ |  |
|  |  |  |  | 65.2 | 4.7 | 0.04 | 6.1 | CE |  |
|  |  |  |  | 66 | 3.18 | 0.06 | 4.86 | 18ZC |  |
| *qST-4-4* | 4 | bin131-bin133 | 46448812-46832786 | 87.1 | 2.76 | -0.04 | 3.33 | CE | （Niu et al. 2013） |
| *qST-5-1* | 5 | bin29-bin31 | 5480651-5715897 | 44.6 | 2.76 | -0.08 | 4.39 | 19GZ |  |
| *qST-5-2* | 5 | bin155-bin158 | 41674961-42084084 | 134.9 | 2.89 | -0.04 | 3.69 | CE | （Niu et al. 2013） |
|  |  |  |  | 135.1 | 3.13 | -0.06 | 4.52 | 20GZ |  |
|  |  |  |  | 135.6 | 3.06 | -0.06 | 4.67 | 18ZC |  |
| *qST-6-1* | 6 | bin4-bin6 | 1104402-1376019 | 3.5 | 2.7 | 0.05 | 3.88 | 20GZ | （Xu et al. 2011） |
| *qST-7-1* | 7 | bin154-bin161 | 35395660-36554645 | 136.4 | 4.06 | 0.06 | 5.75 | 20ZC |  |
|  |  |  |  | 139.9 | 5.37 | 0.05 | 6.99 | CE |  |
|  |  |  |  | 140.8 | 2.69 | 0.05 | 3.85 | 20GZ |  |

Supplementary Table 3 continued

| QTL | Chr^a^ | Interval | Position (bp) | Position (cM) | LOD^b^ | ADD^c^ | PVE (%)^d^ | Env.^e^ | References |
| --- | --- | --- | --- | --- | --- | --- | --- | --- | --- |
| *qST-11-1* | 11 | bin108-bin110 | 28193727-29598942 | 106.1 | 4.57 | -0.07 | 6.7 | 20GZ |  |
| *qST-11-2* | 11 | bin134-bin135 | 33570965-33658063 | 139.1 | 5.99 | 0.09 | 8.92 | 20GZ |  |
| *qST-13-1* | 13 | bin115-bin117 | 27473391-27671175 | 90 | 2.92 | 0.04 | 3.82 | CE | （Hina et al. 2020） |
| *qST-13-2* | 13 | bin132-bin134 | 29638488-30044281 | 99.5 | 4.16 | 0.04 | 5.38 | CE |  |
| *qST-13-3* | 13 | bin143-bin149 | 31125816-32078884 | 103.2 | 3.74 | 0.06 | 5.52 | 19ZC |  |
|  |  |  |  | 104.6 | 3.27 | 0.06 | 5.09 | 19GZ |  |
|  |  |  |  | 105.1 | 3.5 | 0.04 | 4.56 | CE |  |
| *qST-13-4* | 13 | bin210-bin212 | 41247693-41446366 | 164.9 | 3.42 | 0.06 | 5.21 | 18ZC |  |
| *qST-15-1* | 15 | bin30-bin32 | 6130184-7229125 | 35.1 | 2.97 | 0.05 | 4.33 | 19ZC |  |
| *qST-20-1* | 20 | bin38-bin44 | 3056426-4919680 | 44.8 | 2.71 | -0.05 | 3.68 | 20GZ |  |
|  |  |  |  | 46.8 | 4.07 | -0.04 | 5.4 | CE |  |
| *qST-20-2* | 20 | bin67-bin69 | 28731837-32801623 | 55.9 | 5.77 | -0.05 | 7.55 | CE |  |
| *qST-20-3* | 20 | bin81-bin82 | 34185019-34291632 | 62.9 | 2.84 | -0.05 | 4.15 | 19ZC |  |
|  |  |  |  | 63.4 | 3.92 | -0.04 | 5.22 | CE | （Xu et al. 2011） |

^a^Chromosome. ^b^The log of odds (LOD) value at the peak likelihood of QTL. ^d^Phenotypic variation explained. ^e^Environment.

Supplementary Table 4 QTLs identified for 100-seed weight trait in GB13 RIL across different environments

| QTL | Chr^a^ | Interval | Position (bp) | Position (cM) | LOD^b^ | ADD^c^ | PVE (%)^d^ | Env.^e^ | References |
| --- | --- | --- | --- | --- | --- | --- | --- | --- | --- |
| *qHSW-1-1* | 1 | bin12-bin14 | 1696769-1911204 | 9.9 | 2.74 | -0.48 | 3.68 | 18ZC |  |
| *qHSW-3-1* | 3 | bin39-bin41 | 5378498-5821215 | 43.1 | 2.69 | 0.33 | 3.77 | 19ZC |  |
|  |  |  |  | 43.1 | 4.82 | 0.35 | 6.07 | CE |  |
| *qHSW-3-2* | 3 | bin53-bin55 | 9825422-17199876 | 50.1 | 3.2 | 0.37 | 4.41 | 20ZC |  |
|  |  |  |  | 50.1 | 5.43 | 0.37 | 6.81 | CE |  |
|  |  |  |  | 50.6 | 3.68 | 0.38 | 5.12 | 19ZC |  |
| *qHSW-3-3* | 3 | bin66-bin68 | 28943719-29501827 | 56.4 | 3.11 | 0.42 | 4.88 | 19GZ |  |
| *qHSW-4-1* | 4 | bin39-bin41 | 5744948-5838921 | 34.7 | 6.94 | 0.56 | 9.81 | 20ZC |  |
| *qHSW-4-2* | 4 | bin75-bin78 | 9159728-9824768 | 57.5 | 3.25 | 0.37 | 4.68 | 19ZC |  |
|  |  |  |  | 57.5 | 4.18 | 0.33 | 5.28 | CE |  |
|  |  |  |  | 64.4 | 3.75 | 0.59 | 5.34 | 18ZC |  |
| *qHSW-4-3* | 4 | bin152-bin154 | 48442371-48908321 | 104.7 | 2.85 | 0.51 | 4.09 | 18ZC | （Teng et al. 2009；Kato et al. 2014） |
| *qHSW-6-1* | 6 | bin4-bin6 | 1104402-1376019 | 3.5 | 5.09 | 0.55 | 7.18 | 20GZ |  |
| *qHSW-11-1* | 11 | bin72-bin74 | 11002248-11089313 | 71.9 | 2.93 | -0.51 | 4.15 | 18ZC |  |
| *qHSW-11-2* | 11 | bin108-bin110 | 28193727-29598942 | 106.1 | 4.98 | -0.54 | 6.91 | 20GZ |  |
| *qHSW-11-3* | 11 | bin131-bin134 | 32796587-33617864 | 129.5 | 2.56 | 0.32 | 3.33 | 20ZC |  |
|  |  |  |  | 133.1 | 2.99 | 0.42 | 4.9 | 19GZ |  |
|  |  |  |  | 133.1 | 3.41 | 0.31 | 4.41 | CE |  |
|  |  |  |  | 139.1 | 5.76 | 0.59 | 8.26 | 20GZ |  |

Supplementary Table 4 continued

| QTL | Chr^a^ | Interval | Position (bp) | Position (cM) | LOD^b^ | ADD^c^ | PVE (%)^d^ | Env.^e^ | References |
| --- | --- | --- | --- | --- | --- | --- | --- | --- | --- |
| *qHSW-13-1* | 13 | bin115-bin116 | 27473391-27540372 | 89.1 | 3.79 | 0.4 | 5.19 | 20ZC |  |
|  |  |  |  | 90 | 2.91 | 0.39 | 4.34 | 19GZ |  |
|  |  |  |  | 90.1 | 5.69 | 0.38 | 7.21 | CE |  |
| *qHSW-13-2* | 13 | bin132-bin134 | 29638488-30044281 | 97.9 | 3.13 | 0.29 | 4.11 | CE | （Teng et al. 2009） |
| *qHSW-13-3* | 13 | bin233-bin235 | 43433420-45874162 | 188.9 | 4.47 | 0.43 | 6.27 | 19ZC |  |
| *qHSW-14-1* | 14 | bin148-bin150 | 42605675-42819236 | 128.5 | 3.02 | -0.27 | 3.52 | CE |  |
| *qHSW-15-1* | 15 | bin24-bin26 | 4706262-5531628 | 28 | 2.83 | 0.39 | 4.21 | 19GZ |  |
| *qHSW-16-1* | 16 | bin186-bin188 | 36238318-36478110 | 113.9 | 2.77 | -0.4 | 3.81 | 20GZ |  |
| *qHSW-17-1* | 17 | bin40-bin42 | 4938708-5526049 | 44.2 | 3.36 | 0.39 | 5.06 | 19ZC |  |
| *qHSW-17-2* | 17 | bin51-bin53 | 7579564-9011168 | 60.6 | 4.53 | 0.34 | 5.69 | CE | （Teng et al. 2009；Kato et al. 2014） |
| *qHSW-18-1* | 18 | bin29-bin30 | 3254606-3692649 | 18.2 | 3.2 | 0.43 | 4.41 | 20GZ |  |
| *qHSW-20-1* | 20 | bin26-bin28 | 2146582-2308673 | 39 | 3.33 | -0.55 | 4.74 | 18ZC | （Han et al. 2012；Kato et al. 2014） |
| *qHSW-20-2* | 20 | bin42-bin43 | 4610581-4735006 | 45.9 | 2.79 | -0.39 | 4.25 | 19GZ |  |
| *qHSW-20-3* | 20 | bin68-bin73 | 31482024-33222868 | 55.9 | 4.06 | -0.47 | 6.12 | 19GZ |  |
|  |  |  |  | 55.9 | 5.7 | -0.38 | 7.21 | CE |  |
|  |  |  |  | 56.1 | 3.42 | -0.38 | 4.68 | 20ZC |  |
|  |  |  |  | 56.8 | 2.52 | -0.31 | 3.31 | 19ZC |  |
| *qHSW-20-4* | 20 | bin103-bin105 | 35549932-35683070 | 83.6 | 2.6 | 0.48 | 3.49 | 18ZC |  |
| *qHSW-20-5* | 20 | bin123-bin125 | 36748229-36956101 | 96.6 | 2.77 | 0.49 | 3.72 | 18ZC | （Kato et al. 2014） |

^a^Chromosome. ^b^The log of odds (LOD) value at the peak likelihood of QTL. ^d^Phenotypic variation explained. ^e^Environment.
